# Oxidative Burst of Circulating Neutrophils Following Traumatic Brain Injury in Human

**DOI:** 10.1371/journal.pone.0068963

**Published:** 2013-07-24

**Authors:** Yiliu Liao, Peng Liu, Fangyuan Guo, Zhi-Yuan Zhang, Zhiren Zhang

**Affiliations:** 1 Department of Traumatic Surgery, Tongji Hospital, Tongji Medical College, Huazhong University of Science and Technology, Wuhan, People’s Republic of China; 2 Department of Neuropathology, University of Tuebingen, Tuebingen, Germany; 3 Institute of Immunology, Third Military Medical University of PLA, Chongqing, People’s Republic of China; Centro di Riferimento Oncologico, IRCCS National Cancer Institute, Italy

## Abstract

Besides secondary injury at the lesional site, Traumatic brain injury (TBI) can cause a systemic inflammatory response, which may cause damage to initially unaffected organs and potentially further exacerbate the original injury. Here we investigated plasma levels of important inflammatory mediators, oxidative activity of circulating leukocytes, particularly focusing on neutrophils, from TBI subjects and control subjects with general trauma from 6 hours to 2 weeks following injury, comparing with values from uninjured subjects. We observed increased plasma level of inflammatory cytokines/molecules TNF-α, IL-6 and CRP, dramatically increased circulating leukocyte counts and elevated expression of TNF-α and iNOS in circulating leukocytes from TBI patients, which suggests a systemic inflammatory response following TBI. Our data further showed increased free radical production in leukocyte homogenates and elevated expression of key oxidative enzymes iNOS, COX-2 and NADPH oxidase (gp91^phox^) in circulating leukocytes, indicating an intense induction of oxidative burst following TBI, which is significantly greater than that in control subjects with general trauma. Furthermore, flow cytometry assay proved neutrophils as the largest population in circulation after TBI and showed significantly up-regulated oxidative activity and suppressed phagocytosis rate for circulating neutrophils following brain trauma. It suggests that the highly activated neutrophils might play an important role in the secondary damage, even outside the injured brain. Taken together, the potent systemic inflammatory response induced by TBI, especially the intensively increase oxidative activity of circulating leukocytes, mainly neutrophils, may lead to a systemic damage, dysfunction/damage of bystander tissues/organs and even further exacerbate secondary local damage. Controlling these pathophysiological processes may be a promising therapeutic strategy and will protect unaffected organs and the injured brain from the secondary damage.

## Introduction

Traumatic brain injury (TBI) is a leading cause of morbidity and mortality among young adults in developed countries [1]. Following the primary mechanical injury, a secondary or delayed neuronal damage and cell loss develops over a period of hours, days, weeks or months, involving biochemical and molecular changes in the immediate and distant tissues [2]. A robust inflammatory response induced by trauma is a key component of TBI. It involves the activation of glia and neurons as well as cerebral accumulation of monocytes and lymphocytes, which can exacerbate the focal injury and contributes to the secondary or delayed injury at the injury site [3].

Furthermore, TBI can cause bystander damage to organs and tissues unaffected by the original injury as well. Especially, severe TBI often causes multiple organ/tissue dysfunction, damage or failure, therefore complications of TBI are not only restricted to neurological consequences [4], [5], [6]. The inflammatory response following TBI is also not only contained at injury site or in injured brain - neuroinflammation, but involves systemic inflammatory response, which has been reported in patients and in animal models with traumatic injury of CNS, including brain and spinal cord injuries [7], [8]. Previous studies with animal models showed that brain and spinal cord injuries even caused a more intense systemic inflammatory response than general trauma, with ensuing damage to organs, such as lung, liver and kidney [9], [10]. The early, delayed, and systemic effects of acute TBI are the result of inflammatory mediators which initiate systemic inflammatory response, and even subsequent complement deficits and coagulopathy [7]. Inflammatory mediators such as cytokines, free radicals and activation of immune cells are implicated in secondary injury development following brain trauma [11].

As a substantial part of inflammatory response, free radical formation and oxidative damage have been extensively investigated and validated as important contributors to the pathophysiology of acute brain injury. Uncontrolled reactive oxygen chain reactions triggered by secondary injury cascades can feed back into the secondary injury response creating an endless pool of reactive oxygen species (ROS) and the ultimate consequence is massive neuronal death [12], [13]. However, systemic oxidative changes following TBI have not been well understood. Oxidative stress has been linked with onset of systemic inflammation [14] and it’s well known that increased oxidative activity plays important role in pathological process of tissue/organ damages [15].

Following acute TBI, leukocyte counts increase in peripheral blood, since the release of leukocytes from marginal pool stores and the bone marrow into the circulation increases the number of immune cells which are available to cause damage [16], but the destructive characteristics of activated immune cell populations in the circulation is less well understood. Particularly, the immune response after injury is for an important part mediated by neutrophils and systemic inflammatory response following trauma is characterized and reflected by multiple phenotypes of circulating neutrophils [17]. In this present study, inflammatory mediators in circulating immune cells, reactive oxygen species and expression/activity of key oxidative enzymes in immune cell populations, particularly neutrophils, from TBI subjects were investigated and compared to those from uninjured subjects or from a control group who suffered general trauma with no CNS injury and from uninjured subjects.

## Materials and Methods

### Patients

These studies were approved by the ethic committee of Tongji Hospital, Tongji Medical College of Huazhong University of Science and Technology, China, with the ethical approval number of 20120702. All the participants or the next of kin, caretakers, or guardians provided their written informed consent to participate in this study. Venous blood samples were obtained from twenty-eight subjects after consent was obtained according to the World Medical Association outlined in the declaration of Helsinki. Exclusion criteria were polytrauma, a personal or family history of peripheral neuropathy or autoimmune disease, significant cognitive limitations, chronic inflammatory illness, malignant cancer, chronic liver disease, regular medication with anti-inflammatory drugs, diseases of the blood, patients who received blood transfusion, pregnancy or lactation. None of the subjects were treated with steroids or other anti-inflammatory drugs during the two-week period of the study.

The blood samples were taken, when possible, at 6, 12, 24, 48 and 72 hours after the injury in trauma controls (TC) or traumatic brain injury (TBI), as well as at 1 week and 2 week after the injuries. In the TBI group, the patients were formed according to the Glasgow Coma Scale (GCS) score [18] on admission. The lower GCS score indicates more severe disease and higher risk of death. GCS includes only 3 simple tests: eye opening, verbal response, and best motor response. For each variable, the lowest point is 1 while the highest point is 4–6. The total added score of all variables ranges from 3 to 15. Hitherto, good outcome predictions for this patient group can be made as late as after 1week by assessing the recovery/nonrecovery of the GCS score to greater than 8 [19]. Patients with a GCS score ≤8 were considered as severely brain-injured, and patients with a GCS score >8 were considered as moderately brain-injured. If a subject enrolled in the study required a blood transfusion, then no further samples were obtained from that patient. The TBI subjects were diagnosed by computed tomographic (CT) scanning. The trauma controls had significant fractures of long bones, ribs, pelvis or vertebrae without CNS injury. All TBI and trauma control subjects were thoroughly examined to exclude multi-system trauma and had computerized tomography scans of the thorax, abdomen and pelvis to exclude other associated injuries. The patients showed no evidence of systemic infection or local infection during the 2 week period of the study. All blood samples were taken under sterile condition and were analyzed in a laminar flow hood in a tissue culture laboratory. All the samples for the background measurements were from the same donors. Details of these patients are in Table 1.

**Table 1:** Description of involved subjects.

Subject group	Case#	GCS	Age	Sex	Mechanism of trauma
TBI	#1	7	54	F	MVA
	#3	3	58	F	MVA
	#4	10	47	M	Fall
	#5	12	38	F	MVA
	#7	3	24	M	MVA
	#10	7	41	F	MVA
	#13	14	58	F	Fall
	#14	6	62	F	MVA
	#15	10	25	M	Fall
	#16	14	28	F	Fall
	#18	8	52	F	MVA
	#22	7	49	M	MVA
Trauma control		**Fracture location**			
	#2	Double upper limbs	36	F	Fall
	#6	Right lower limbs	35	M	MVA
	#8	Right upper limbs	26	F	MVA
	#9	Left clavicle, left 4^th^,5^th^ and 6^th^ rib	47	F	MVA
	#11	Right lower limbs and right scapula	53	F	Fall
	#12	Double lower limbs	26	M	MVA
	#17	Pelvis	24	F	Fall
	#19	2^nd^ lumbar	58	M	Fall
	#20	Left lower limbs	51	F	MVA
	#21	Mandible	43	M	Fall
Uninjured	#1		26	F	
	#2		26	F	
	#3		27	F	
	#4		26	F	
	#5		29	M	
	#6		30	M	

TBI, Traumatic brain injury; GCS, Glasgow Coma Scale; MVA, Motor vehicle accident; M, male; F, female.

### Oxidative Activity Analysis

Oxidative activity was detected by staining with dihydrorhodamine123 (DHR123, Santa Cruz Biotechnology Inc, CA), a non-fluorescent agent that is converted by cellular oxidation to the fluorescent dye rodamine123 (R123). Heparinized whole blood samples (100 µl) from uninjured, trauma control and TBI subjects were incubated with DHR123 (1.0 µM) in 400 µl RPMI Medium1640 at 37°C in sterile conditions; the leukocytes were then isolated by ammonium chloride lysis of red blood cells. Neutrophils, monocytes and lymphocytes were initially gated by their characteristic forward and side scatter profiles, which represent size and granularity of the cells, respectively. Cells in these gates were then analyzed for fluorescence intensity. Cell-associated R123 fluorescence within the three populations was determined using a FACScalibur flow cytometer (Becton Dickinson, San Jose, CA). Background fluorescence (in samples incubated without R123) was subtracted from total fluorescence to obtain the value of oxidative activity from each individual sample.

### Phagocytosis Assay

Phagocytosis assay was performed as described previously [20], [21], with minor modulation. Alexa-labeled E. coli were prepared by the following method. The bacteria of logarithmic growth phase were washed 2 times with PBS and then resuspended in PBS at a concentration of 0.5×10^9^/ml. Fluorescein Alexa 488 (Santa Cruz Biotechnology Inc, CA) was prepared as a 5 mg/ml solution in the dimethyl sulfoxide. The Alexa-solution was then added to the bacterial suspension and made the concentration of Alexa to 5 ug/ml. This suspension was incubated at 37°C in darkness for 2 hr followed by being washed two times in PBS. Bacteria were fixed with 1%Paraformaldehyde for 30 minutes, then resuspended in the same PBS and were aliquoted at 1×10^9^/ml and stored at 4°C in darkness.

The bacteria suspension (15 ul) were mixed with heparinized whole blood samples (100 µl) from uninjured, trauma control and TBI subjects, then they were incubated for 15 min at 37°C and stored on ice for 10 min to stop the reaction. The leukocytes were then isolated by ammonium chloride lysis of red blood cells. Phagocytosis of fluorescein Alexa-labeled E. coli is quantified and expressed as percentage of neutrophils and monocytes through flow cytometry analysis.

### Free Radical Assay

Reactive oxygen species in the homogenates, same as for the DHR123 analysis, were detected by their oxidative conversion of the non-fluorescent 2′-7′-dichlorofluorescein-diacetate (DCFH-DA, Beyotime, PRC) to the fluorescent 2′-7′-dichlorofluorescein (DCF). The fluorescence was determined using a flow cytometer.

### Western Blotting to Detect iNOS and COX-2 and NADPH Oxidase (gp91^phox^) Expression

Standard methods for immunoblotting and analysis were used to detect iNOS, COX-2 and NADPH oxidase (subunit gp91^phox^), employing the antibodies listed above for immunocytochemistry. The leukocyte homogenates contained mostly neutrophils and lymphocytes, and a smaller number of monocytes, as confirmed by the flow cytometry studies above. As the oxidative activity of lymphocytes is small in comparison to that of neutrophils, the findings in the assays of the leukocyte homogenates can be attributed mostly to neutrophils.

Proteins were extracted from each blood sample to do the electrophoresis and loaded onto polyacrylamide gel, separated by sodium dodecyl sulfate–polyacrylamide gel electrophoresis (SDS–PAGE) and transferred to polyvinylid-ene difluoride (PVDF) membranes (0.45 lm pore size, Millipore, Mississauga, ON, Canada). Leukocytes were lysed by RIPA lysis buffer (P0013B, Beyotime, Shanghai, China) for 2 minutes, 10,000 rpm/for 5 minutes. The protein concentration of the supernatant was measured by a BCA assay kit at 564 nm (BCA method, P0009, Beyotime, Shanghai, China). Using a standard curve to calculate the protein concentration 75 ug protein per lane and sample was loaded. The membranes were first blocked with 5% non-fat powdered milk and then incubated with an anti-iNOS antibody (1: 700, BS1186, Bioworld Technology Inc, Louis Park, MN), or anti-COX-2 (1∶700, BS1076, Bioworld Technology Inc, Louis Park, MN), anti-gp91^phox^ (subunit of NADPH oxidase) (1∶600, BS9035, Bioworld Technology Inc, Louis Park, MN), followed by incubation with horseradish peroxidase-conjugated sheep anti-rabbit secondary antibodies (1∶40000, BA1054, Boster Biological Technology LTD. Wuhan, China). The primary antibody was incubated over night at 4°C, and the second antibody was incubated for 2 hours. Five washing steps were conducted between antibody incubation steps, using 1 M Trisbuffered saline-Tween (TBST) solution.

Signal detection was facilitated with enhanced chemiluminescence (ECL kit, ThermoFisher, USA). Immunoreactive bands were scanned by an imaging densitometer (Bio-Rad GS-700 Imaging Densitometer) and the results were quantified using Multi-Analyst software (Bio-Rad). Densitometric values were normalized for protein loading using the β-actin as a loading control and for local background. Molecular weights of the proteins detected were determined using known molecular weight protein standards (Bio-Rad Prestained Precision Protein Standards).

### Immunocytochemical Staining of Blood Smears

A smear was made from leucocyte of each sample and fixed in acetone overnight. The slides containing the smears were kept at −20°C until they were used. Blood smears were immunostained with antibodies for or against the oxidative enzymes inducible nitric oxide (iNOS, 1∶1000, Bioworld Technology Inc, Louis Park, MN) and cyclooxygenase-2 (COX-2, 1∶1000, Bioworld Technology Inc, Louis Park, MN), to the transcription factor Nuclear Factor-κB p65 (NF-κB, 1∶1000, Bioworld Technology Inc, Louis Park, MN), and to the catalytic subunit of nicotinamide adeninedinucleotide phosphate (NADPH) oxidase (gp91^phox^, 1∶1000, Bioworld Technology Inc, Louis Park, MN). All stainings were performed using the same protocol, steps of smearing, fixing, closing, washing, coloring and alcohol dehydration were conducted at room temperature, and others were at 37°C. After quenching endogenous peroxidase activity by inhibition with 3% H_2_O_2_ for 10 minutes, the smears were incubated with the primary antibodies described above for 2 hour. They were then washed in 0.01 M Trisbuffered saline (TBS) and incubated with Polymer Helper for 20 minutes. After rinsing in TBS, polyperoxidase-anti-mouse/rabbit IgG (PV-9000, ZSGB Biotechnology, Beijing, China) was applied for 25 minutes. The sections were then washed in TBS and immunoreactivity was visualized after incubation in a glucose-diaminobenzidine-nickel solution for 5 min. The stained smears were rinsed in TBS, dehydrated through a gradient of ethanol (75% for 2 min, 80% for 2 min, 95% for 2 min, anhydrous ethanol for 2 min, anhydrous ethanol for 2 min at room temperature), cleared, and cover slipped with Cytoseal mountant (ZLI-9555, ZSGB Biotechnology, Beijing, China). TBS was substituted for the primary antibody on control smears in each reaction. A portion of the blood sample from six uninjured subjects was activated ex vivo with the pro-inflammatory chemotactic peptide methionyl-leucyl-pheny-lalanine (fMLP, 2.5 nM) for 30 min at room temperature (24°C) prior to preparing the smear to ascertain responses to a known in flammatory agent. For quantitative analysis of optical density of immunoreactive product, three smears from each subject (four subjects each group) were used and leukocyte was analyzed using Image-Pro Plus6.0 software (Olympus, Tokyo, Japan) to obtain a measure of positive area and optical density.

### Quantification of Cytokines and Acute Phase Proteins in Plasma

Cytokine levels in plasma were measured using the following enzyme-linked immunosorbent assay kits: IL-6 and tumor necrosis factor-a (TNF-a) (E-EL-H0102 for IL-6 and E-EL-H0109 for TNF-a, Wuhan Elabscience Biotechnology, Wuhan, China) were determined with a semiautomatic chemoluminescence-based enzyme-linked immunosorbent assay system (microplate reader, Tecan Group Ltd, Salzburg, Austria). Collected whole blood with heparin as anticoagulant was centrifuged for 15 minutes at 1200 rpm at 2–8°C. The supernatant was collected for the following assay. The micro ELISA plates provided in the kits had been pre-coated with an antibody specific to IL-6/TNF-α. Standards or samples are then added to the appropriate micro ELISA plate wells and combined to the specific antibody. Then a biotinylated detection antibody specific for IL-6/TNF-α and Avidin-Horseradish Peroxidase (HRP) conjugate was added to each micro plate well and incubated. Free components were washed away. The substrate solution was added to each well. Only those wells that contain IL-6/TNF-α, biotinylated detection antibody and Avidin-HRP conjugate appeared blue in color. The enzyme-substrate reaction was terminated by the addition of a sulphuric acid solution and the color turned yellow. The optical density (OD) was measured spectrophotometrically at a wavelength of 450 nm. The OD value was proportional to the concentration of IL-6/TNF-α. The calculation of concentrations of IL-6/TNF-α in the samples was done by comparing the OD value of the samples to the standard curve, which was prepared accordingly to the kit manual, using the software (curve expert 1.3).

C-reactive protein (CRP) levels were measured immunoturbidimetrically using analyser CRP-LATEX(II)X2 (600714, Denka Seiken Co. Ltd, Japan,). Whole blood was centrifuged for 15 minutes at 1200 rpm at 2–8°C and the supernatant was collected. The microcentrifuge tubes containing the periopaper strips and plastic vials containing serum were transferred to the lab for immunoturbidimetric analysis. Serum was used undiluted. The normal value of CRP is <3 mg/L. The CRP-concentration was analyzed by the fully automatic biochemical analyzer. Light through the solution was refracted when encounter the CRP-complex and the intensity of scattered light proportional to the content of the complex. Concentration of CRP was automatically calculated by the analyzer.

### Statistical Analysis

Data are expressed as the mean ± standard deviation [18]. Differences among groups were established using one-way analysis of variance (ANOVA) and Fisher’s protected t-tests or by a Student’s t test when only two groups were compared. Statistical significance was established at P<0.05.

## Results

### Inflammatory Cytokine/Molecule Concentrations in Plasma

**Figure 1:**
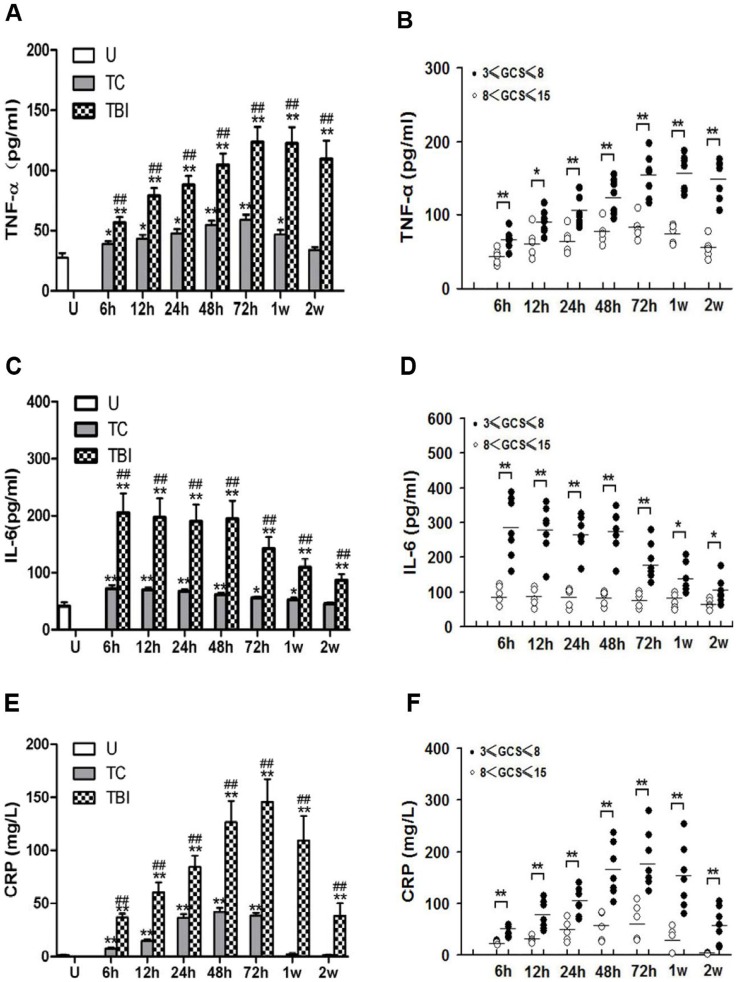
Inflammatory marker concentrations in plasma. Mean changes in TNF-α (A, B), IL-6 (C, D) and CRP (E, F) in plasma after injury in trauma controls and traumatic brain injury subjects are presented. Concentration is expressed as mean ± SE values and samples are plotted as histograms at times encompassing 6 h-2 weeks after injury. Uninjured subjects (n = 6) (U, open bars) had low concentration of TNF-α, IL-6 and CRP. The concentration of TNF-α, IL-6 and CRP in trauma control (n = 10) (TC, grey bars) subject was significantly increased at 6 h-1 week (for CRP only to 72 hours) and in traumatic brain injury (n = 12) (TBI, black bars) subject was significantly increased at 6 h-2 weeks after injury. In contrast, increases of TNF-α, IL-6 and CRP after TBI were greater than those of TC subjects at throughout the entire observation period. (B, D, F) The patients with traumatic brain injury were divided into severe (STBI, black spots) (n = 5) and moderate (MTBI, white spots) (n = 7) brain injury. The concentration of TNF-α, IL-6 and CRP in TBI subject with STBI and MTBI were plotted as graphs at times encompassing 6 h-2 weeks after injury. Increases of the concentration of TNF-α, IL-6 and CRP from STBI subjects were greater than those from MTBI subjects throughout the entire observation period (from 6 h to 2 weeks after injury). **P<0.01; *P<0.05, significantly different from uninjured by Fisher’s protected t tests. ##P<0.01; #P<0.05, significantly different from trauma controls by Fisher’s protected t tests. **P<0.01; *P<0.05, significantly different from MTBI by Fisher’s protected t tests.

Plasma concentrations of TNF-α, IL-6 and CRP in subjects without injury or following control trauma and traumatic brain injury were determined. Uninjured subjects (n = 6) had low concentrations of TNF-α (29.4±2.1 pg/ml) and IL-6 (42.6±2.1 pg/ml), and nearly undetectable CRP (1.4±0.3 mg/L) (Fig. 1A, C, E). The concentrations of TNF-α and IL-6 in trauma control subjects (n = 10) were significantly increased from 6 hours (6 h) until 1 week (1 w) following injury (TNF-α: 1.3, 1.5, 1.6, 1.9, 2.0, 1.6-fold to uninjured controls; IL-6: 1.7, 1.7, 1.6, 1.4 1.3 1.2-fold to uninjured controls for 6 h, 12 h, 24 h, 48 h, 72 h and 1 w after trauma), of CRP only from 6 hours until 72 h (5.3, 10.5, 26.2, 30.1, 27.6-fold for 6 h, 12 h, 24 h, 48 h, 72 h after trauma). In TBI subjects (n = 12), however, concentrations of all the three molecules were significantly increased at all time points assessed (6 h - 2 w after injury). Furthermore, increases of TNF-α (1.9, 2.7, 3.0, 3.5, 4.1, 3.9, 3.6-fold to uninjured controls for 6 h-2 w after trauma), IL-6 (4.5, 4.3, 4.1, 4.2, 3.2, 2.5, 1.9-fold to uninjured controls) and CRP (23.6, 37.0, 51.9, 25.7, 87.4, 62.7, 21.9-fold to uninjured controls) after TBI were significantly greater than those of trauma control subjects at 6 h –2 w following injury (compared using t-tests, P<0.05 for all the results of TNF-α, IL-6 and CRP, from 6 h to 2 w). In TBI subjects, TNF-α concentration increased steadily from 6 h to 72 h and reached the plateau from 72 h to 2 w; IL-6 concentration reached the peak right already at 6 h and slowly decreased after 48 h; CRP concentration increased steadily since 6 h, reached the peak at 72 h and slowly decreased thereafter.

The patients with TBI were further divided into severe (STBI, 3≤ GCS score ≤8; n = 5) and moderate (MTBI, 8< GCS score ≤15; n = 7) brain injury groups. Increases of the concentrations of TNF-α, IL-6 and CRP from STBI subjects were significantly greater than those from MTBI subjects throughout the entire observation period (6 h to 2 w after injury) (compared using t-tests, P<0.05 for all the results of TNF-α, IL-6 and CRP, from 6 h to 2 w) (Figures 1B, D, F).

### Free Radical Production in Leukocyte Homogenates

**Figure 2:**
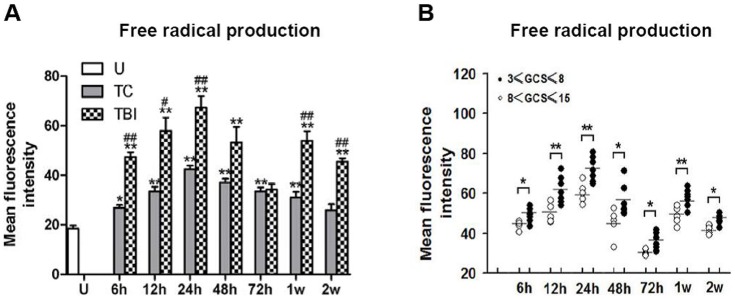
Free radical production in leukocyte homogenates. The presence of free radicals in the leukocytes was estimated by the conversion of DCFH to DCF in homogenates from uninjured (U) subjects and from trauma controls (TC) and traumatic brain injury (TBI) subjects at times ranging from 6 h to 2 weeks after injury. (A) Uninjured subjects (U, open bars) had a few DCF. DCF concentrations increased significantly in TC (grey bars) and TBI (TBI, black bars) subjects at most times assessed. Changes in leukocytes from TBI subjects were greater than those from TC subjects at 6 h, 12 h, 24 h, 1 week and 2 weeks after injury. (B) Futher, mean fluorescence intensity in leukocyte homogenates of six uninjured subjects, twelve TBI subjects with five MTBI (white circles) and seven STBI (black circles) were plotted as a scatter gram. Increases of free radical in leukocytes from STBI subjects were greater than those from MTBI subjects throughout the entire observation period (from 6 h to 2 weeks after injury). **P<0.01; *P<0.05, significantly different from uninjured by Fisher’s protected t tests. ##P<0.01; #P<0.05, significantly different from trauma controls by Fisher’s protected t tests. **P<0.01; *P<0.05, significantly different from MTBI by Fisher’s protected t tests.

The presence of free radicals in the leukocytes was estimated with the fluorescent DCF. Uninjured subjects had a low concentration of DCF (18.7±1.6) (Figure 2A). DCF concentrations increased significantly in trauma controls and TBI subjects at almost all the time points assessed, except after 2 weeks for the trauma controls (compared using t-tests, P<0.05 for all the time points, except for after 2 w) (Figure 2A). Maximal increases were detected at 24 h after injury when DCF (41.9±1.7) was increased by 2.2 fold in the trauma controls and by 3.6 fold in the TBI subjects (67.3±6.0). Increase of DCF concentration from TBI subjects was significantly greater than those from trauma control subjects at 6 h, 12 h, 24 h, 1 w and 2 w after injury (compared using t-tests, P<0.05 for all these time points) (Figure 2A).

Further determination of free radical productions between different severities of brain injury revealed significantly increases of free radical in leukocytes from severe injured (STBI) subjects than those from moderate injured (MTBI) subjects at all the time points assessed (STBI vs. MTBI: 1.2-, 1.2-, 1.2-, 1.3-, 1.2-, 1.2-, 1.1-fold for 6 h to 2 w respectively, compared using t-tests, P<0.05 for all time points from 6 h to 2 w after injury) (Figure 2B).

### Expression of iNOS, COX-2 and NADPH Oxidase (gp91^phox^) in Leukocytes

**Figure 3:**
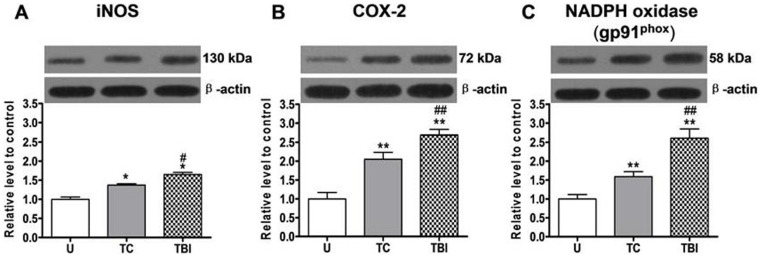
Expression of iNOS, COX-2 and NADPH oxidase (gp91^phox^) in leukocyte homogenates. From uninjured (U), trauma controls (TC) and traumatic brain injury (TBI) subjects, expression of iNOS, COX-2 and NADPH oxidase (gp91^phox^) in leukocyte homogenates at 24 h after injury are determined by western blot assay. Upper blots are typical western blots of enzyme expression in each group. Lower blots are β-actin expression demonstrating equal protein loading of gels for each enzyme. Bottom bar graphs show quantification of expression by densitometry, with enzyme expression expressed as a percent of β-actin density ± SE (n = 5 all groups). iNOS, COX-2 and NADPH oxidase (gp91^phox^) expression in both TC and TBI subject was significantly increased, while the expression was greater after TBI than in trauma control subjects. **P<0.01; *P<0.05, significantly different from uninjured by Fisher’s protected t tests. ##P<0.01; #P<0.05, significantly different from trauma controls by Fisher’s protected t tests.

Expression of iNOS, COX-2 and NADPH oxidase (gp91^phox^) (Figure 3A, B, C respectively) in leukocytes from all the subjects was determined using western blot assay. Low level expression of all these proteins was readily detected in leukocyte homogenates of uninjured subjects. At 24 h after injury in both trauma control or TBI subjects, iNOS, COX-2 and NADPH oxidase (gp91^phox^) expressions increased significantly (1.6-fold, 2.1-fold and 1.4-fold to uninjured controls, respectively in trauma controls; 2.6-fold, 2.7-fold and 1.7-fold to uninjured controls, respectively in TBI subjects) (Figure 3), compared to the uninjured subjects, while the expressions were greater following TBI than in trauma control subjects (compared using t-tests, P<0.05 for all these comparisons) (Figure 3).

Spatial expression of iNOS, NADPH oxidase (gp91^phox^), COX-2, and the proinflammatory transcription factor NF-κB (nucleus) in leukocytes were further determined using immunostaining on blood smears from uninjured, trauma controls and TBI subjects at 24 h after injury. As activation control, one group of leukocytes from uninjured subjects was treated with with fMLP (U+fMLP).

**Figure 4:**
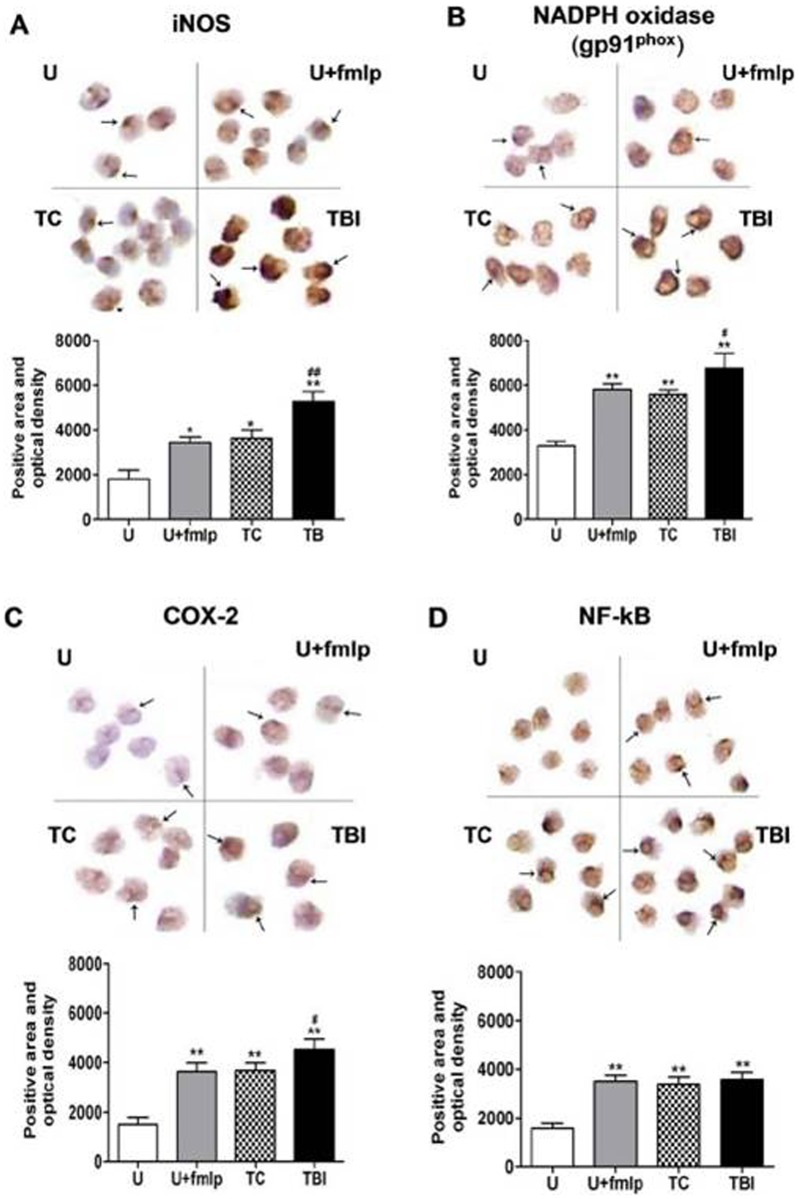
Expression of iNOS, NF-kB, COX-2 and NADPH oxidase (gp91^phox^) in leukocytes on blood smears. From uninjured (U), uninjured with fMLP activation (U+fMLP) and trauma controls (TC) and traumatic brain injury (TBI) subjects, expression of iNOS, NF-κB, COX-2 and NADPH oxidase (gp91^phox^) in leukocytes subjects at 24 h after injury are determined with immunostaining on blood smears. Blood smears were immunostained for the NF-κB (nucleus) and the oxidative enzymes (cytoplasm). Photomicrographs at the top of A-D are typical examples of immunostained cells amplified 400 times and the arrows indicate the positive expression. Blood smears were analyzed by Image-Pro plus 6.0 software to obtain a measure of positive area and optical density. Bar graphs in lower panels of A–D depict mean positive area and optical density ± SE for each group (n = 5 subjects/group). After treatment of blood samples with fMLP and in TC and TBI subjects, expression of iNOS, NF-kB, COX-2 and NADPH oxidase (gp91^phox^) all increased significantly when compared to values from uninjured subjects. iNOS, COX-2 and NADPH oxidase (gp91^phox^) expression was greater after TBI than in trauma control subjects. **P<0.01; *P<0.05, significantly different from uninjured by Fisher’s protected t tests. ##P<0.01; #P<0.05, significantly different from trauma controls by Fisher’s protected t tests.

Slight immunoreactivity of iNOS, NADPH oxidase (gp91^phox^), COX-2 and NF-kB, was detected in leukocytes from uninjured subjects (Figures 4A, B, C, D respectively), revealing low level expression of them. In leukocytes from trauma control and TBI subjects at 24 h after injury, or from uninjured subjects but treated with fMLP, obviously stronger immunoreactivity of iNOS, NADPH oxidase (gp91^phox^), NF-kB and COX-2 were observed (compared using t-tests, P<0.05 for all these comparisons). Further quantitative image analysis also proved significantly increased staining area and optical intensity.

Active NF-κB was mostly in the nucleus with fainter expression in the cytoplasm (Fig. 4D). Staining area and optical intensity of NF-κB had a significant, over 2-fold increase after treatment with fMLP, as well as in samples from trauma controls and TBI subjects, compared to that in the uninjured subjects, but there was no difference between fMLP stimulation, trauma controls and TBI groups (compared using t-tests, P<0.05 for all these comparisons to U control and P>0.05 for all comparisons between each other) (Fig. 4).

Immunostaining of NADPH oxidase (gp91^phox^), iNOS and COX-2 in leukocytes was confined to the cytoplasm and at low levels in leukocytes of uninjured subjects. Their staining area and optical intensity significantly increased following treatment with fMLP and in samples from trauma controls and TBI subjects (gp91^phox^: 1.8-fold, 1.7-fold and 2.1-fold to uninjured controls respectively; iNOS: 1.8-fold, 2.0-fold and 2.9-fold to uninjured controls respectively; COX-2: 2.5-fold, 2.5-fold and 3.0-fold to uninjured controls respectively). Furthermore, staining area and optical intensity of iNOS, NADPH oxidase (gp91^phox^) and COX-2 from the TBI subjects was greater than in trauma control subjects (compared using t-tests, P<0.05 for all these comparisons).

### Increased Leukocyte Counts and Oxidative Burst of Neutrophils Following Brain Injury

24 hours following injury, increased leukocyte counts were observed in circulation from trauma control subjects and brain injured subjects, compared to uninjured controls. Circulating counts of neutrophils and monocytes were also increased. Especially, neutrophil counts were dramatically increased following brain injury, even more than that from trauma controls. However, monocyte counts were only slightly increased in circulation of trauma controls and brain injured subjects (Data are listed in Table 2).

**Table 2:** Subjects at 24 h after injury.

	Leukocyte(×10^9.^L^−1^)	Neutrophil(×10^9.^L^−1^)	Monocyte(×10^9.^L^−1^)
Uninjured	6.42±0.58	4.82±0.68	0.38±0.09
Trauma control	13.58±1.04 *	11.37±0.78 *	0.84±0.08 *
TBI	18.68±0.76 * #	17.13±0.57 * #	1.04±0.07 *

* P<0.05, significantly different from uninjured by Fisher’s protected t tests. #P<0.05, significantly different from trauma controls by Fisher’s protected t tests.

**Figure 5:**
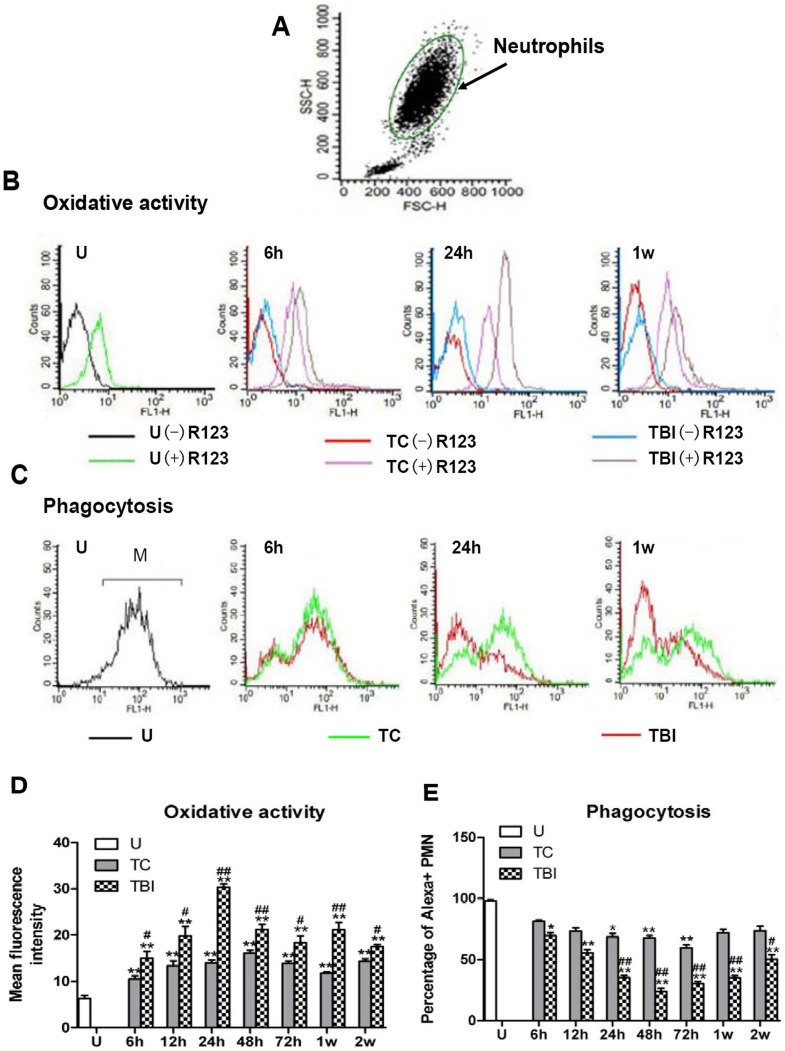
Oxidative activity and phagocytosis rate of neutrophils after injury in trauma controls and TBI subjects. (A) Example of gating of a blood sample for flow cytometry using physical characteristics of granularity (side scatter) and size (forward scatter). Neutrophils constituted the largest population. (B) Histograms plotting the number of cells vs. intensity of R123 fluorescence (log scale) produced by incubation of blood samples with DHR123 from uninjured subjects (U, green lines) and from trauma controls (TC, purple lines) and TBI (grey lines) subjects at 6 h, 12 h, 24 h, 48 h, 72 h, 1 week and 2 weeks after injury (only representative images from u, 6 h, 24 h and 1 w are shown in the figure). Samples incubated without DHR123 revealed background fluorescence for uninjured (black line), TC (red lines) and TBI (blue lines) subjects. Both TC and TBI subjects had increases in fluorescence due to oxidative burst in neutrophils, while the TBI subjects increased more than trauma control subjects. (C) Neutrophils of uninjured subjects engulfed Alexa-labeled E. coli, the proportion of neutrophils with the labeled fluorescent analyzed by flow cytometry was phagocytosis rate M. Graphs plotting the number of cells vs. intensity of Alexa fluorescence (log scale) from uninjured subjects (U, black line) and from TC (green lines) and TBI (red lines) subjects at 6 h, 12 h, 24 h, 48 h, 72 h, 1 week and 2 weeks after injury. Both TC and TBI subjects had decreases in phagocytosis rate of neutrophils, while the TBI subjects decreased more than trauma control subjects. (D and E) Oxidative activity is expressed as mean ± SE values of R123 fluorescence (after subtraction of background fluorescence) and phagocytosis rate is expressed as mean ± SE values of the proportion of neutrophils with the labeled fluorescent. All samples are plotted as histograms at times encompassing 6 hours – 2weeks after injury. Uninjured subjects (U, open bars) had low oxidative activity. Oxidative burst in TC (grey bars) subject was significantly increased and the increase is even greater in neutrophils from TBI subjects (black bars) throughout the entire observation period (from 6 h to 2 weeks after injury). Neutrophils had substantial oxidative activity at 24 h, 48 h and 1 week after TBI. Uninjured subjects (U, open bars) had high phagocytosis rate. Phagocytosis in TC (grey bars) subject was significantly decreased in circulating neutrophils at 24 h, 48 h and 72 h. In contrast, phagocytosis rate decreased significantly at 6 h–2 weeks after TBI (black bars) in neutrophils from 6 h to 2 week in monocytes. Decreases after TBI are greater than those of trauma control subjects in neutrophils from 12 h to 2 weeks. **P<0.01; *P<0.05, significantly different from uninjured by Fisher’s protected t tests. ##P<0.01; #P<0.05, significantly different from trauma controls by Fisher’s protected t tests.

Oxidative status of particular cell populations in circulation is further determined by flow cytometry (FACS). At first, cell populations were defined/characterized by granularity (side scatter, SS) and size (forward scatter, FS), neutrophils were selected by live gates (FS versus SS) as previously described [22], [23]. An example figure of a blood sample gating is shown in the figure 5A (Figure 5A). Neutrophils constituted the largest population whereas monocytes made up a smaller population. FACS results are presented as histograms plotting the number of cells vs. intensity of R123 fluorescence produced by incubation of blood samples with DHR123 (Figure 5B).

At all the time points assessed, 6 h, 12 h, 24 h, 48 h, 72 h, 1 w and 2 w after injury, both trauma control and TBI subjects had increases in intensity of R123 fluorescence of neutrophils due to oxidative burst, while significantly stronger increases TBI subjects (2.4, 3.2, 4.8, 3.4, 2.9, 3.4, 2.8-fold to uninjured controls respectively) were observed compared to trauma control subjects (1.7, 2.1, 2.2, 2.6, 2.2, 1.9, 2.3-fold to uninjured controls respectively) (compared using t-tests, P<0.05 for all these comparisons) (Figure 5D). Neutrophils had substantial oxidative activity at 24 h, 48 h and 1 w after TBI (Figure 5D).

### Decreased Phagocytosis of Neutrophils Following TBI

Phagocytosis rate of neutrophils from uninjured subjects, after injury in trauma controls and traumatic brain injury subjects were determined by flow cytometry. Neutrophils were introduced with Alexa-labeled E. coli and the proportions of neutrophils with the labeled fluorescent were analyzed by FACS. The example graphs plotting the number of neutrophil cells vs. intensity of Alexa fluorescence (log scale) from uninjured subjects, trauma controls and traumatic brain injury subjects is presented in the Figure 5C.

Uninjured subjects had high phagocytosis rate, nearly 100% (99.1±0.5%). Both trauma control and TBI subjects had decreases in phagocytosis rate of neutrophils, while the TBI subjects decreased significantly more than trauma control subjects (compared using t-tests, P<0.05 for all the time points). Phagocytosis in trauma control subject was significantly decreased at 24 h, 48 h and 72 h (68.1±4.6%, 65.8±3.8%, 59.6±3.1% respectively, compared using t-tests, P<0.05 for the 3 time points). In contrast, phagocytosis rate decreased significantly throughout the entire observation period (6 h–2 w after TBI: 68.5±4.4%, 55.7±4.6%, 35.0±2.6%, 22.2±4.3%, 29.7±3.2%, 35.3±2.8%, 48.4±6.0%) in TBI subjects (compared using t-tests, P<0.05 for all the time points). Decreases after TBI were greater than those of trauma control subjects in neutrophils from 24 h to 2 w after injury (compared using t-tests, P<0.05 for all the time points). Neutrophils had the lowest levels of phagocytosis at 48 h (22.2±4.3%) and 72 h (29.7±3.2%) after TBI.

## Discussion

Our present study reported increased plasma level of inflammatory markers TNF-α, IL-6 and CRP, dramatically increased circulating leukocyte counts, particularly neutrophils counts, and elevated expression of TNF-α and iNOS in circulating leukocytes from TBI patients, suggesting a systemic inflammatory response following TBI. Intensively increased free radical production in leukocyte homogenates and elevated expression of key oxidative enzymes iNOS, COX-2 and NADPH oxidase (gp91^phox^) in circulating leukocytes, even significantly greater than in control subjects with regular trauma, indicated an intense induction of oxidative burst following TBI. Furthermore, flow cytometry assay proved neutrophils as the largest population in circulation after TBI and showed significantly up-regulated oxidative activity and suppressed phagocytosis rate for circulating neutrophils following brain trauma, suggesting the highly activated neutrophils might play an important role in pathological process of the secondary damage, even outside the injury brain.

Severe TBI often causes multiple organ/tissue dysfunction, damage, failure or even death, which may be a consequence of systemic inflammatory response [4], [5], [6]. A characterizing initiator of systemic inflammatory response is the entry of these inflammatory mediators from lesional site into circulation [24], [25]. Massive increase of local inflammatory mediators including cytokines and free radicals is well known following TBI which may eventually lead to complications of systemic hyperinflammation, followed by immunosuppression, multi-organ dysfunction syndrome (MODS) and even death, days to weeks after trauma [7], [11], [26].

In our present study, increased plasma levels of inflammatory TNF-α, IL-6 and CRP were detected as early as 6 hours after general trauma or TBI, their elevated level stayed until the end of our observation (2 w) in TBI subjects. Previous studies also reported elevated levels of inflammatory markers in blood following brain trauma, including IL-1, IL-6, IL-8, TNF-α and furthers [27], [28]. Most of them, especially IL-6, can be generated in the injury site of brain, then enter blood circulation through the damaged BBB and affect other tissue or organ functions [29]. IL-6 is the dominant cytokine in inflammation, produces the acute phase response and is involved in regulating the levels of other cytokines [30]. TNF-α is a proinflammatory and procoagulative cytokine, and is associated with the development of systemic inflammatory response syndrome (SIRS) and further organ/tissue damage [31]. Elevated CRP levels was reported during SIRS, especially following trauma and its production is related to the severity of organ dysfunction [32], [33]. Significant up-regulaion of iNOS on circulating leukocytes was further observed, that indicated a substantially greater capacity of NO production, which may contribute to the secondary injury cascade follwing injury, both in lesion site and other organs [34].

The post-injury inflammatory response is mainly mediated by innate immune response and characterized not only by up-regulation of inflammatory mediators, but also by activation of immune cells [35], [36]. Leukocytosis is characterizing marker of systemic inflammatory response to physical trauma and is associated with focal and systemic consequences [37]. In the present study, we observed increased leukocyte counts in circulation following injury, especially dramatically elevated neutrophils counts, constituting the largest population, which may suggest a more dominant role of neutrophil activation during the systemic inflammatory response. The innate immune response after injury is for an important part mediated by neutrophils, they are essential in the first line of defense, the amount of neutrophils can be rapidly increased by release from bone marrow [38]. During systemic inflammatory response, primed circulating neutrophils are prone to home and to become activated in the tissue/organ encountering additional inflammatory stimuli, and may eventually lead to multiple organ dysfunction/damage, such as damage, dysfunction and even failure of lung and liver [5], [10], [39], [40], [41], [42].

Increased oxidative activity is another essential features of leukocyte activation during systemic inflammatory response [5]. Free radical formation and oxidative damage are considered as important contributors to the pathophysiology and one of the best validated secondary injury mechanisms of acute central nervous system injury, especially TBI [12], [13]. Further, processes of oxidative stress in connection with continued systemic inflammatory response may promote the development of multiple organ failure (MOF) [43]. Animal studies have shown that neutrophils can generate reactive oxygen, nitrosyl radicals and oxidative enzymes [44], [45]. Oxidative burst was previously reported in neutrophils and/or monocytes after spinal cord injury of human [8] and rat [10]. In our present study, significantly elevated free radical production in leukocyte homogenates was observed as early as 6 hours, and until the end of our observation period of TBI and control injuries (2 weeks). Western blot of leukocyte homogenates and immunostaining on blood smears further proved increased expression of key enzymes in the induction of oxidative burst at 24 hour following TBI. Further FACS analysis revealed the intense induction of oxidative burst in neutrophils, the major population of increased circulating leukocytes, throughout the entire observation period. Increased oxidative activity and up-regulation of oxidative enzymes are important characters of immune cell activation and all these different parameters regarding increased oxidative burst, increased free radical production and expression of key enzymes correlated well in our study. Robust oxidative activity of these activated immune cells contributes to secondary inflammatory damage of focal lesion and surviving tissue/organs [12], [13]. Neutrophils are main and first cell populations to infiltrate into lesional sites and into other organs, the increased counts, inflammatory activation and oxidative activity may greatly contribute to not only local damage of lesional sites but also secondary damage to unaffected bystander organs or tissues [17], [25], [46].

Meanwhile, significantly decreased phagocytic activity of neutrophils were observed in TBI subjects throughout the entire observation period, and greater than that of trauma control since 24 hours after injury. This observation is in accordance with a previous report regarding spinal cord injury [47], and may be a result of a regulatory mechanism to minimize the deleterious effects of increased neutrophil burst activity.

Interestingly, increased oxidative burst, increased free radical production, up-regulation of key oxidative enzyme expression and even elevated plasma levels of inflammatory cytokines in TBI subjects are greater than that from the general trauma controls. It indicates usually greater inflammatory response following TBI than general injuries outside of CNS. Similar phenomena has been reported previously in TBI [9] and in spinal cord injury patients and animals [8], [40], that injury of CNS can cause an intense systemic inflammatory response. However, the exact mechanism is not clear yet, might be due to loss of crucial feedback control of immune function by injury/trauma of CNS. Furthermore, our data show that severe TBI subjects had stronger oxidative burst and higher plasma levels of inflammatory mediators than moderate TBI subjects, which is in accordance with previous studies with rodents suggesting a strong correlation of injury severity with the inflammatory response [48]. However, only a weak correlation could be observed for human TBI until now, probably due to multifactorial aetiology of injury and inter-patient variability [17], [49].

Taken together, TBI causes a potent systemic inflammatory response including up-regulated inflammatory mediators and activation of immune cells in circulation, as well as increased oxidative burst, elevated free radical production, up-regulation of key oxidative enzyme expression of leukocytes, particularly of the major population of neutrophils. That may lead to systemic damage, dysfunction/damage of bystander tissue/organs and even further exacerbate secondary local damage. Controlling these pathophysiological processes may be a promising therapeutic strategy, will protect unaffected organs and the injured brain from the secondary damage and might help to decrease mortality rate for TBI patients.
