# Correction: Oxidative Burst of Circulating Neutrophils Following Traumatic Brain Injury in Human

**DOI:** 10.1371/annotation/73ce45d3-4478-4c06-a4d2-3274b6222bae

**Published:** 2013-09-23

**Authors:** Yiliu Liao, Peng Liu, Fangyuan Guo, Zhi-Yuan Zhang, Zhiren Zhang

Multiple funding organizations and grants were incorrectly given in the Funding Statement. In addition, multiple funding organizations and grants were incorrectly omitted from the Funding Statement. The Funding Statement should read:

"This present research was partially supported by funding as follows:

To Zhiren Zhang- 1, Chongqing City of China, Natural Science Foundation Project of Chongqing Science and Technology commission (No. 2010BB5025, http://www.ctin.ac.cn/Class.aspx?clsId=425), 2, State Education Ministry of China Scientific Research Foundation for the Returned Overseas Chinese

Scholars (No. 2012-44, http://fund.cscse.edu.cn/Login.aspx).

To Yuliu Liao- 1, State Education Ministry of China Scientific Research Foundation for the Returned Overseas Chinese Scholars (No. 2009-1001, http://fund.cscse.edu.cn/Login.aspx), 2, Hubei Province of China Hubei Provincial Natural Science Foundation (No. 2011CDB543, http://jhsb.hbstd.gov.cn/).

The funders had no role in study design, data collection and analysis, decision to publish, or preparation of the manuscript."

